# Clinical Effects of MOTOmed Intelligent Exercise Training Combined with Intensive Walking Training on the Rehabilitation of Walking, Nerve and Lower Limb Functions among Patients with Hemiplegia after Stroke

**DOI:** 10.12669/pjms.38.5.5259

**Published:** 2022

**Authors:** Yajing Hu, Jingjing Tian, Xiaoliang Wen, Can Lu, Ningning Tian

**Affiliations:** 1 Yajing Hu, School of Nursing and Health, Hebei College of Science and Technology, Baoding 071000, Hebei, China; 2 Jingjing Tian, School of Nursing and Health, Hebei College of Science and Technology, Baoding 071000, Hebei, China; 3 Xiaoliang Wen, Department of Neurology, The Hospital of 82nd Group Army PLA, Baoding 071000, Hebei, China; 4 Can Lu, School of Nursing and Health, Hebei College of Science and Technology, Baoding 071000, Hebei, China; 5 Ningning Tian, Department of Neurology, The Hospital of 82nd Group Army PLA, Baoding 071000, Hebei, China

**Keywords:** MOTOmed intelligent exercise training, Intensive walking training, Stroke, Hemiplegia, Walking function, Nerve function, Lower limb function

## Abstract

**Objectives:**

To investigate the clinical effects of MOTOmed intelligent exercise training combined with intensive walking training on the rehabilitation of walking, nerve and lower limb functions among patients with hemiplegia after stroke.

**Methods:**

Randomized controlled trial was used in this study. Fifty-two patients with hemiplegia after stroke treated in 82nd Army Group Military Hospital from February 2017 to February 2018 were selected as the subjects and randomly divided into the control group and the observation group, each with 26 cases. The control group underwent intensive walking training, and the observation group underwent MOTOmed intelligent exercise training on the control group basis. Both groups’ rehabilitation of walking function, nerve function and lower limb function were observed.

**Results:**

Both groups had significantly increased FAC score and 10-m maximum walking speed (*P* < 0.05), and the observation group had significantly higher results than those of the control group (*P* < 0.05); both groups had significantly higher FMA scores than before treatment (*P* < 0.05), and the observation group had significantly higher scores than those of the control group (*P* < 0.05); both groups after two months of treatment had significantly increased NGF, NT-3 and BDNF (*P*<0.05), and the observation group had significantly higher levels than those of the control group (*P*<0.05).

**Conclusion:**

MOTOmed intelligent exercise training combined with intensive walking training can significantly improve the walking function, nerve function and lower limb function among patients with hemiplegia after stroke.

## INTRODUCTION

Stroke is an acute cerebrovascular disease, including ischemic and hemorrhagic, caused by brain tissue damage due to abnormal blood supply of cerebral blood vessels. Most stroke occurs in people over 40 years old, more males than females, and has higher morbidity, mortality and disability rates.^1^ Hemiplegia is a common symptom of acute cerebrovascular disease. When the hemiplegia occurs, patients will have movement disorders of the upper and lower limbs, facial muscles and lower lingual muscles on the same side. Effective treatment for stroke has been unavailable. Clinically, more than 50% of the patients surviving after stroke have been found to have limb dysfunction of varying degrees^2^, seriously affecting their ability to daily living, normal standing and upright walking, and the recovery of lower limb function is particularly important for patients with stroke.^3^ MOTOmed intelligent exercise training is a new type of rehabilitation equipment containing various training modes. It can record and feedback the training progress and recovery of patients in real time, assist the rehabilitation therapist to timely determine the next training intensity and frequency, maximize the improvement of limb movement ability, and help physical function recovery.^4^ Therefore, in this study, MOTOmed intelligent exercise training combined with intensive walking training was applied to help patients with hemiplegia after stroke to recover their walking function, neurological function and lower limb function, so as to provide effective reference for clinical treatment.

## METHODS

Randomized controlled trial was used in this study. A total of 52 patients with post-stroke hemiplegia diagnosed and treated in 82nd Army Group Military Hospital from February 2017 to February 2018 were selected as the research subjects. All met the diagnostic criteria for stroke described in the Key Points for Diagnosis of Various Cerebrovascular Diseases.^5^ The sample size required for each group is calculated by the formula:


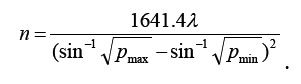


### Ethical Approval:

The study was approved by the Institutional Ethics Committee of Hebei College of Science and Technology on February 10, 2017 (Ref. No. 2017-11), and written informed consent was obtained from all participants.

### Inclusion Criteria:

- Meeting the relevant diagnostic criteria above;
- Being diagnosed as stroke by CT or MRI;
- Being diagnosed with first onset and one side of the lower limbs impaired;
- Having poor walking ability but not significantly affected cognitive ability;
- Being approved by the Medical Ethics Committee and having the ICF completed.

### Exclusion criteria:

- Being companied by severe organ dysfunction such as heart, liver and kidney;
- Having recurrent stroke;
- Being accompanied by severe mental disorder.

All patients were randomly divided into the control group and the observation group using random number table method. The control group had 26 cases, including 15 males and 11 females, ranging from 44 to 73 years old, with an average age of (56.97±10.24) years, and a course of disease ranging from one to six years, with an average course of (3.13±1.21) years, 10 of cerebral infarction, 14 of cerebral hemorrhage, 15 of left hemiparesis and 13 of right hemiparesis; the observation group had 26 cases, including 16 males and 10 females, ranging from 45 to 77 years old, with an average age of (56.22±10.37) years, and a course of disease ranging from 10 months to six years, with an average course of (3.15±1.19) years, 9 of cerebral infarction, 16 of cerebral hemorrhage, 12 of left hemiparesis and 15 of right hemiparesis. Both groups showed no statistically significant difference in general information such as gender, age, course of disease, and disease type (*P*>0.05), and they were comparable.

### Methods:

The control group received routine rehabilitation trainings, including: correct prone position; turning over; lying in bed: bridge-style movement, hugging knees, and self-movement with fingers crossed; movement of limbs and joints; training of hemiplegic upper limbs; standing and walking; daily self-care skills. The observation group had MOTOmed intelligent exercise training (MOTOmed Viva 2, RECK company, Germany) on the control group basis. Patients were required to take a sitting position and an appropriate mode (including passive training, assisted training, active resistance training) was selected according to their lower limbs motor function for the pedaling and circular movement. Both the observation group and the control group received 20min training every time, once a day, 6 times a week, for 2 consecutive months.

### Observation Index:

Walking function: Both groups before and after the rehabilitation training had the walking ability evaluation by using functional ambulation category scale (FAC), and the 10-m maximum walking speed was also measured. Classification criteria of FAC: Grade 0: unable to stand or walk; Grade-1: walk within 10 meters with the help of others indoors; Grade-2: walk 20 meters indoors under supervision of others; Grade 3: walk more than 50 meters indoors independently; Grade 4: continue to walk more than 100 meters, up and down 10 steps; Grade 5: continue to walk more than 200 meters, can independently up and down the steps.

### Lower limbs function:

Both groups before and after the rehabilitation training had the lower limbs motor function assessment by using Fugl-Meyer assessment (FMA). FMA is an ordinal scale with 3 scores for each item. Subjects were given 0 points for failing to complete an item, one point for partial completion, and two points for complete completion. However, there were only two scores for reflex activity, with the presence and absence of reflex activity being two and 0 respectively. The five areas assessed by the FMA were motor function (the highest score was 66 for the upper extremities and 34 for the lower extremities), sensory function (maximum 24 points), balance (maximum 14 points), range of motion (maximum 44 points), and joint pain (maximum 44 points).

### Neural function:

Both groups before and after the rehabilitation training had test for nerve growth factor (NGF), brain-derived neurotrophic factor (BDNF) and neurotrophin-3 (neurotrophin-3, NT-3) in peripheral venous blood by using ELISA.

### Statistical Analysis:

In this study, the statistical software SPSS 20.0 was used to analyze and process data; counting data was expressed as *Rate* (%); x^2^- test was used for inter-group comparison; and the measurement data were expressed as *mean ± standard deviation* (x̄±S); *t*-test was used for intra-group or inter-group comparison, and *P* < 0.05 indicated that the difference was statistically significant.

## RESULTS

Both groups before treatment had no significant difference in FAC score and 10-m maximum walking speed (*P*>0.05); both groups after treatment had significantly increased FAC score and 10-m maximum walking speed (*P* < 0.05), and FAC score and 10-m maximum walking speed in the observation group were significantly higher than those in the control group, with statistical significance (*P* < 0.05). Table-I.

**Table I:** Comparison of walking function between the 2 groups after treatment (χ¯±S).

Group	FAC (scale)		*t*	*p*	10m maximum walking speed (m/min)		*t*	*p*

	Before	After	-	-	Before	After	-	-
Control group (n=26)	1.63±0.79	2.98±0.76	6.279	0.000	30.23±12.36	47.68±12.57	5.047	0.000
Observation group (n=26)	1.59±0.86	3.92±0.64	11.083	0.000	32.34±12.27	56.28±15.47	6.182	0.000
*t*	0.175	4.824	-	-	0.618	2.200	-	-
*p*	0.862	0.000	-	-	0.540	0.032	-	-

Both groups had no significant difference in FMA scores before treatment (*P*>0.05); both groups after 2 months of treatment had significantly higher FMA scores than those before treatment (*P*<0.05), and the FMA score in the observation group was significantly higher than that of the control group, with statistical significance (*P* < 0.05). Table-II.

**Table II:** Comparison of FMA scores between the 2 groups before and after treatment (χ¯±scores).

Group	Cases	Before	After	*t*	*p*
Observation group	26	9.69±2.43	16.84±2.88	9.675	0.000
Control group	26	9.67±2.56	12.03±2.34	3.470	0.001
*t*	-	0.029	6.609	-	-
*p*	-	0.977	0.000	-	-

Both groups had no statistically significant difference in the levels of NGF, NT-3, and BDNF before treatment (*P*>0.05); both groups after 2 months of treatment had significantly higher NGF, NT-3, and BDNF, and these in the observation group were significantly higher than those in the control group (*P* < 0.05). Table-III

**Table III:** Comparison of nerve factor levels before and after treatment in the 2 groups (χ¯±ng/L).

Group	Time	n	NGF	Nt-3	BDNF
Observation group	Before	26	6.72±0.28	7.69±0.44	9.72±0.30
	After	26	12.63±0.94	11.78±0.94	19.36±2.32
	*t/P*		30.725/0.000	20.094/0.000	21.012/0.000
Control group	Before	26	6.76±0.25	7.73±0.82	9.67±0.31
	After	26	8.75±0.82	9.16±0.87	12.93±2.01
	*t/P*		11.837/0.000	6.099/0.000	8.173/0.000
Comparison of the 2 groups before treatment	*t/P*		0.543/0.589	0.219/0.827	0.591/0.557
Comparison of the 2 groups after treatment	*t/P*		15.860/0.000	10.430/0.000	10.681/0.000

## DISCUSSION

Stroke has become the leading cause of death in China. It is common that the embolus in the blood vessel wall of the brain falls off and blocks the artery, as well as the cerebral hemorrhage. Thrombolytic therapy is often applied in clinical practice, but patients are often accompanied by different degrees of limb disorders. Research results have shown^6^-^8^ the central nervous system of the brain is still partially compensatory after injury, which can be re-established through system training and re-learning; and it is expected to improve patients’ limb dyskinesia and improve the quality of life after discharge based on its plasticity. Kimberley TJ et al.^9^ have showed correct rehabilitation training can up-regulate the gene expression of nerve growth factor, affect neurotransmitter transmission, and promote the recovery of nerve function. Therefore, apart from drug administration, rehabilitation training has become the most important treatment for patients with stroke. Studies have shown that gait speed and walking endurance improved in patients six months after stroke after repeated motor function and balance training.^10^ Cardiopulmonary function, muscle strength, balance and walking ability were significantly improved in patients with chronic stroke after resistance training and aerobic training.^11^-^13^

MOTOmed intelligent exercise training is powered by the engine and includes three modes of passive training, assisted exercise, and active resistance training. The operation mode can be adjusted artificially according to patients’ lower limbs movement, having the advantages of repeatability and pertinence.^14^ It can promote blood circulation in the lower limbs, prevent muscle atrophy, and improve the motor function, so as to help patients re-establish normal exercise, and regain confidence in treatment.^15^ Studies have shown^16^,^17^ that MOTOmed intelligent exercise training can reduce the spasm of the lower limbs of patients with stroke, expand the range of joint motion, improve the exercise ability of the lower limbs, strengthen the muscles of the lower limbs, and improve their walking stability. The results of this study show that, the FAC score, 10-m maximum walking speed and the FMA score in the observation group were significantly higher than those in the control group, with statistical significance (*P* < 0.05). This indicates that MOTOmed intelligent motor training can significantly improve patients’ walking function and lower limb motor function through repeated movements of patients’ lower limbs, which were similar to the results of previous studies. Passive training mode may be adopted for patients with complete loss of exercise ability, which was driven by the engine to passively complete seated treadmill training. If the patient does not completely lose the ability to exercise, but has part of the muscle strength, and can partially resist resistance and self-gravity, the assisted training mode may be adopted to assist the patient to complete the trample exercise and enhance the strength of the residual muscle group. If the patient can carry out resistance exercise, the active resistance training mode may be adopted, in which the patient completes the trampling exercise on the premise of resistance, and improves the existing muscle strength while utilizing the residual muscle strength, so that the patients can recover their autonomous activity shortly.^18^ Meanwhile, the biofeedback function of MOTOmed intelligent exercise training can timely deal with patients’ sudden lower limb muscle spasm during the training process, and the motor operation direction will slowly reverse until the spasm is released.^19^ In our study, both groups after two months of treatment had significantly higher NGF, NT-3, and BDNF, and these in the observation group were significantly higher than those in the control group (*P* < 0.05). This indicated that MOTOmed intelligent motor training can significantly improve the neurological function of patients and promote the recovery of motor function, which were similar to the study results of Skvortsova VI, et al.^20^ The results of this study have shown that MOTOmed intelligent exercise training can significantly improve patients’ walking function and nerve function by repetitive movement of their lower limbs, and promote the recovery of the motor function, indicating that MOTOmed intelligent exercise training can enhance the coordination and stability of lower limb joints, improve the walking speed, also continuously stimulates the recovery of the movement and sensation of the limbs, promote the recovery of muscle strength and muscle tension, and prevent muscle atrophy; it can also help the brain synapses to re-establish connections and promote the nutritional repair of damaged nerves.

### Limitations of this study:

The number of subjects included in this study is limited, so the conclusions drawn may not be very convincing. In addition, there are few studies on MOTOmed intelligent exercise in previous studies, which proves the innovation of this study, but also makes the discussion of this study insufficient.

## CONCLUSION

For patients with hemiplegia after stroke, MOTOmed intelligent exercise training combined with intensive walking training can significantly improve the patients’ walking function and speed, promote the nutritional repair of nerve factors and improve nerve damage, significantly improve lower limb movement disorders, and help patients recover their motor function of the lower limbs, improve treatment confidence, providing an effective reference for rehabilitation training of patients with hemiplegia after stroke.

### Authors’ Contributions:

**YH & NT:** Designed this study, prepared this manuscript, are responsible and accountable for the accuracy and integrity of the work.

**CL:** Collected and analyzed clinical data.

**JT & XW:** significantly revised this manuscript.
